# Association of polymorphisms in the aldosterone-regulated sodium reabsorption pathway with blood pressure among Hispanics

**DOI:** 10.1186/s12919-016-0054-5

**Published:** 2016-10-18

**Authors:** Bamidele O. Tayo, Liping Tong, Richard S. Cooper

**Affiliations:** Department of Public Health Sciences, Stritch School of Medicine, Loyola University Chicago, Maywood, IL 60153 USA

## Abstract

**Background:**

Whereas genome-wide association study (GWAS) has proven to be an important tool for discovery of variants influencing many human diseases and traits, unfortunately its performance has not been much of all-around success for some complex conditions, for example, hypertension. Because some of the existing effective pharmacotherapeutic agents act by targeting known biological pathways, pathway-based analytical approaches could lead to more success in discovery of disease-associated variants. The objective of the present study was to identify functional variants associated with blood pressure in the aldosterone-regulated sodium reabsorption pathway using the simulated and real blood pressure phenotypes provided for Genetic Analysis Workshop 19.

**Methods:**

The present analysis included 1942 samples with exome sequencing data and for whom blood pressure phenotypes were available. Because only odd-numbered autosomes were available, we restricted analysis to 127 quality-controlled single-nucleotide polymorphisms from the aldosterone-regulated sodium reabsorption pathway. We performed pathway-based association analysis using appropriate regression models for single variant, haplotype and epistasis association analyses. To account for multiple comparisons, statistical significance was empirically derived by permutation procedure and Bonferroni correction.

**Results:**

The topmost pathway-based association signals were observed in *PRKCA* gene for diastolic blood pressure (DBP), systolic blood pressure (SBP), and mean arterial pressure (MAP) in both real and simulated data. The associations remained significant (*P* <0.05) after multiple testing corrections for the number of genes. Similarly, the pathway-based 2-locus epistasis analysis indicated significant interactions between *INSR* and *PRKCG* for SBP and MAP; *INS* and *PIK3R2* for DBP; *PIK3CD* and *ATP1B2* for hypertension in the real data set. We also observed significant within-gene interactions in *INSR* for SBP, DBP, and hypertension in the simulated data set.

**Conclusion:**

The findings from this study show that pathway-based analytical approach targeting known biological pathways can be useful in identification of disease-associated variants that are otherwise undetectable by GWAS. The approach takes advantage of the assumption of nonindependence of variants within and across pathway genes which leads to reduced penalty of multiple testing and thus less-stringent statistical significance threshold.

## Background

Genome-wide association study (GWAS) has proved to be a useful tool in the discovery of genetic variants associated with many complex diseases and traits [1]. Unfortunately, the level of success in variants discovery by GWAS for some complex human diseases, such as elevated blood pressure or hypertension, has been very low. In fact, variants so far discovered through GWAS collectively explain only a small fraction of the known heritability for any of the diseases [2–5].

Given what is known about the biology of these diseases and traits, it is suspected that important variants with moderate to large effect sizes remain to be identified; this is commonly referred to as the “missing heritability” [2, 3, 6, 7]. The explanations for missing heritability include the postulation that it could lie in regulatory rare variants, functional variants, structural variants, gene-by-gene or gene-by-environment interactions [8–11]. It has also been suggested that multiple small-effect variants, which are individually undetectable with the statistical power of GWAS, additively contribute to the missing heritability [12–14]. Another explanation is that the current estimates of total heritability may have been significantly inflated by the effects of epistasis [15]. The search for missing heritability has witnessed application of various approaches including pathway-based analysis of common, less frequent, and rare variants [16–18]; analysis of correlated traits using summary statistics from GWAS [19]; and analytical procedures that accommodate mixture of effects on the traits [20]. Because some of the existing effective pharmacotherapeutic agents for blood pressure control act by targeting specific biological pathways and these pathways are less represented in the GWAS-identified variants [1, 21–24], analytical approaches that focus on known biological pathways rather than on the entire genome could lead to discovery of some of the variants linked to “missing heritability” in association studies.

Consequently, the main objective of the present study was to perform pathway-based association analysis to identify blood pressure phenotypes–associated functional variants in the aldosterone-regulated sodium reabsorption pathway using whole exome sequence data provided for Genetic Analysis Workshop 19 (GAW19). The aldosterone pathway was chosen because it is one of the known target biological pathways for pharmacological control of hypertension. We hypothesize that functional genetic variant in the pathway influences susceptibility to blood pressure elevation.

## Methods

Analyses were based on the unrelated data set of human whole exome sequence data plus the simulated and real phenotypes data as provided for GAW19 and described by Almasy et al [25].

### Study subjects and phenotypes

The study samples included 1943 adult Hispanic subjects, that is, 1021 type 2 diabetes cases and 922 controls from the San Antonio Family Heart Study, San Antonio Family Diabetes/Gallbladder Study, Veterans Administration Genetic Epidemiology Study, and the Investigation of Nephropathy and Diabetes Study family component (HA) [26–29]; and the Starr County, Texas (HS) [30, 31] studies. Available study variables included sex, age, diastolic blood pressure (DBP), systolic blood pressure (SBP), and use of antihypertensive medication. Of the 1943 subjects, only 1850 had complete data on study variables.

We analyzed both the simulated blood pressure phenotypes in the “SIMPHEN.1” data set and the real blood pressure phenotypes in the “T2D-GENES_P1_Hispanic_phenotypes” data set. Outcome variables included in the analysis are DBP, SBP, pulse pressure (PP) (defined as *PP = SBP − DBP*), mean arterial pressure (MAP) (defined as *MAP = DBP+[PP/3])*, and hypertension (defined as blood pressure ≥140/90 mm Hg or use of antihypertensive medication). Sex, age, and age-squared were treated as covariates in the analysis.

### Genotype data

Whole exome sequence data were provided on 11 odd-numbered autosomes. The genotypes used in the present analysis were based on NALTT (number of nonreference alleles for each individual thresholded) as provided in the variant call format (VCF) files. We used the software BCFtools (http://samtools.github.io/bcftools/bcftools.html) to extract data on biallelic (single nucleotide and deletion/insertion) variants and then recoded the genotypes from 0/1/2 to ACGT using the information on both the reference and alternate alleles for each variant. The quality control (QC) of the genotype data was carried out using the software PLINK [32]. Of the 1,765,688 total available variants, 1,711,766 were biallelic. We excluded 136,233 variants with missing genotypes greater than 10 % and 1,529,240 variants with minor allele frequency of less than 1 %. Rare variants were excluded because the focus of the analysis was on common and less-frequent variants and also because the sample size was too small for single-variant analysis of rare variants. Another 1238 variants that failed Hardy-Weinberg equilibrium test at *p* <0.001 were excluded. One sample with missing genotypes of greater than 10 % was excluded. The final quality-controlled genotype data set was made up of 1942 samples and 45,055 biallelic variants. Principal component analysis was performed using all 45,055 variants and the first of 10 components was extracted and included in association analysis to control for population stratification. Only the 1850 subjects with complete data on blood pressure phenotypes were included in association analysis.

### Aldosterone-regulated sodium reabsorption pathway genes

The aldosterone-regulated sodium reabsorption pathway was defined using KEGG PATHWAY Database (http://www.genome.jp/kegg/pathway.html). The pathway comprises of 39 genes located across 14 autosomes and the X chromosome. Twenty-two genes were on the 11 odd-numbered autosomes available for the present analysis (Table 1). Annotation of variants was done using the SeattleSeq Annotation (http://snp.gs.washington.edu/SeattleSeqAnnotation138/index.jsp). Of the 45,055 biallelic variants that passed QC, a total of 127 were in the aldosterone-regulated sodium reabsorption pathway. With the exception of the *SFN* gene, each of the 22 genes on the odd-numbered autosomes had at least 1 variant available for analysis.

**Table 1:** Aldosterone-regulated sodium reabsorption pathway genes available in the data set

Gene name	Definition	KEGG Orthology No.	Chromosome	GRCh38 location
*PIK3CD*	Phosphatidylinositol-4,5-bisphosphate 3-kinase, catalytic subunit delta	K00922	1	9629889 to 9729114
*SFN*	Stratifin	K06644	1	26863142 to 26864456
*PIK3R3*	Phosphoinositide-3-kinase, regulatory subunit 3 (gamma)	K02649	1	46040140 to 46133036
*ATP1A1*	ATPase, Na+/K+ transporting, alpha 1 polypeptide	K01539	1	116372180 to 116404774
*ATP1A2*	ATPase, Na+/K+ transporting, alpha 2 polypeptide	K01539	1	160115730 to 160143591
*ATP1A4*	ATPase, Na+/K+ transporting, alpha 4 polypeptide	K01539	1	160151562 to 160186977
*ATP1B1*	ATPase, Na+/K+ transporting, beta 1 polypeptide	K01540	1	169106709 to 169132722
*PIK3CB*	Phosphatidylinositol-4,5-bisphosphate 3-kinase, catalytic subunit beta	K00922	3	138652698 to 138834938
*ATP1B3*	ATPase, Na+/K+ transporting, beta 3 polypeptide	K01540	3	141876628 to 141926540
*PIK3CA*	Phosphatidylinositol-4,5-bisphosphate 3-kinase, catalytic subunit alpha	K00922	3	179148114 to 179235137
*PIK3R1*	Phosphoinositide-3-kinase, regulatory subunit 1 (alpha)	K02649	5	68215756 to 68301821
*PIK3CG*	Phosphatidylinositol-4,5-bisphosphate 3-kinase, catalytic subunit gamma	K00922	7	106865278 to 106908978
*INS*	Insulin	K04526	11	2159779 to 2161209
*FXYD2*	FXYD domain containing ion transport regulator 2	K01538	11	117820075 to 117828092
*KCNJ1*	Potassium inwardly-rectifying channel, subfamily J, member 1	K04995	11	128838014 to 128867373
*ATP1B2*	ATPase, Na+/K+ transporting, beta 2 polypeptide	K01540	17	7650936 to 7657771
*PIK3R5*	Phosphoinositide-3-kinase, regulatory subunit 5	K02649	17	8878916 to 8965712
*PRKCA*	Protein kinase C, alpha	K02677	17	66302642 to 66810744
*INSR*	Insulin receptor	K04527	19	7112255 to 7294328
*PIK3R2*	Phosphoinositide-3-kinase, regulatory subunit 2 (beta)	K02649	19	18153178 to 18170533
*ATP1A3*	ATPase, Na+/K+ transporting, alpha 3 polypeptide	K01539	19	41966476 to 41994276
*PRKCG*	Protein kinase C, gamma	K02677	19	53882213 to 53907647

### Association analysis

Using the simulated and real phenotypes, we fitted additive linear (for DBP, SBP, PP, MAP) and logistic (for hypertension status) regression models for each outcome variable with the variant as explanatory variable coded as dosage of the minor allele. Sex, age, age-squared, and first principal component were included as covariates. The software PLINK [32] was used for the association analysis by implementing the set-based tests. All the 127 variants in the pathway were considered as a set. The test involved iterative steps that included: (a) for each variant, we determined which other variants were in linkage disequilibrium above a certain threshold *R* ^*2*^ and eliminated other variants with values above the threshold; (b) performed single-variant association analysis and selected up to *N* variants with *p* values below *P*, starting with the most significant one; (c) from the subset of variants, we calculated set-statistic as the mean of the single-variant statistics; (d) permuted the data set 5000 times and repeated steps (b) and (c) for each permuted data set; (e) calculated empirical *p* value as the number of times the permuted set–statistic exceeded the original data set–statistic. Software default values of 0.5, 0.05, and 10 were used for the parameters *R* ^*2*^, *P,* and *N*, respectively. For each outcome variable, haplotype and epistasis association analyses were also done. The epistasis association involved all pairwise combinations of the 127 variants and their interaction. We also performed Bonferroni correction for multiple testing using number of testing as equal to the number of genes in the pathway. This was based on the assumption of nonindependence of variants in the pathway genes.

## Results

**Fig. 1:**
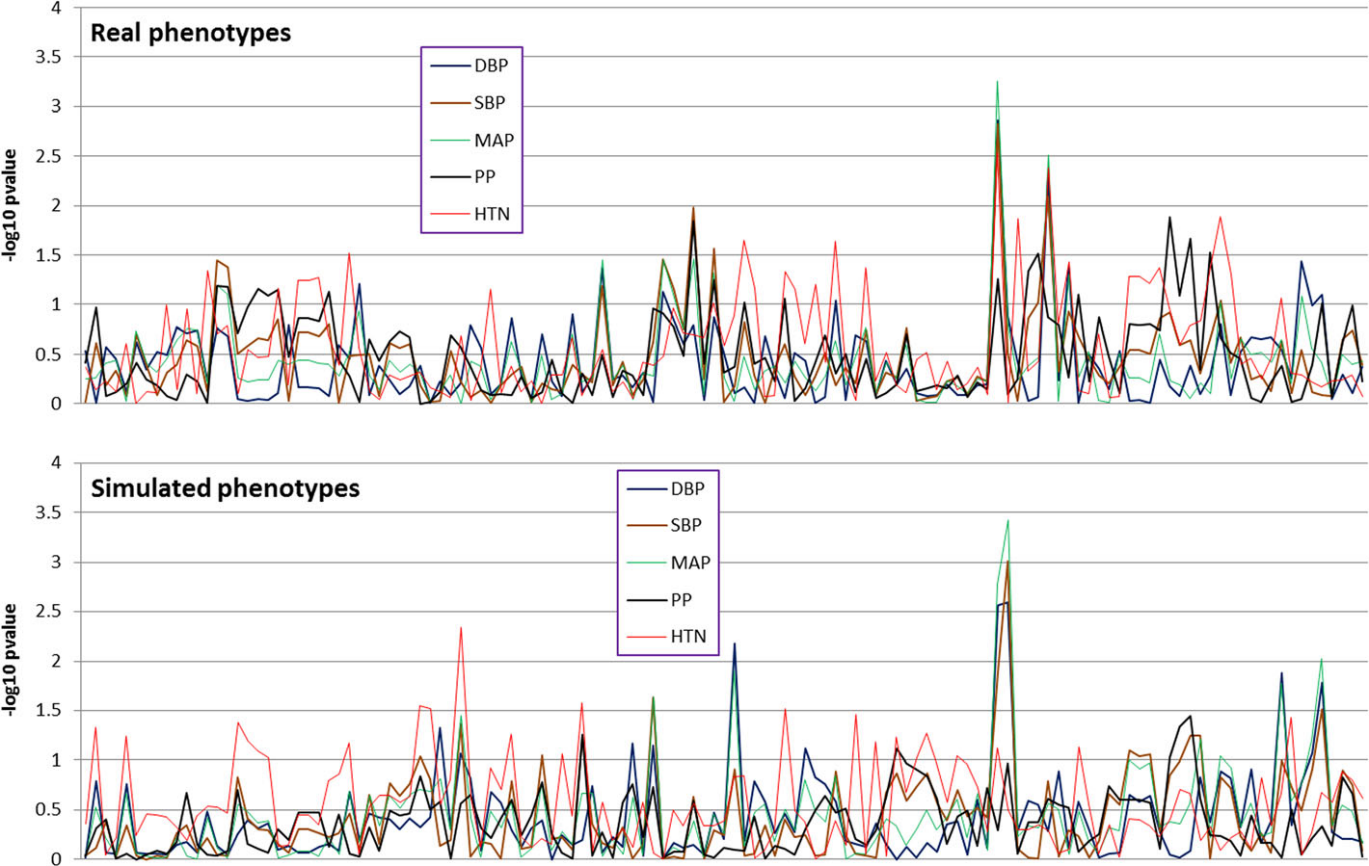
Distributions of single single-nucleotide polymorphism association signals for real phenotypes *(top)* and simulated phenotypes *(bottom)*

**Fig. 2:**
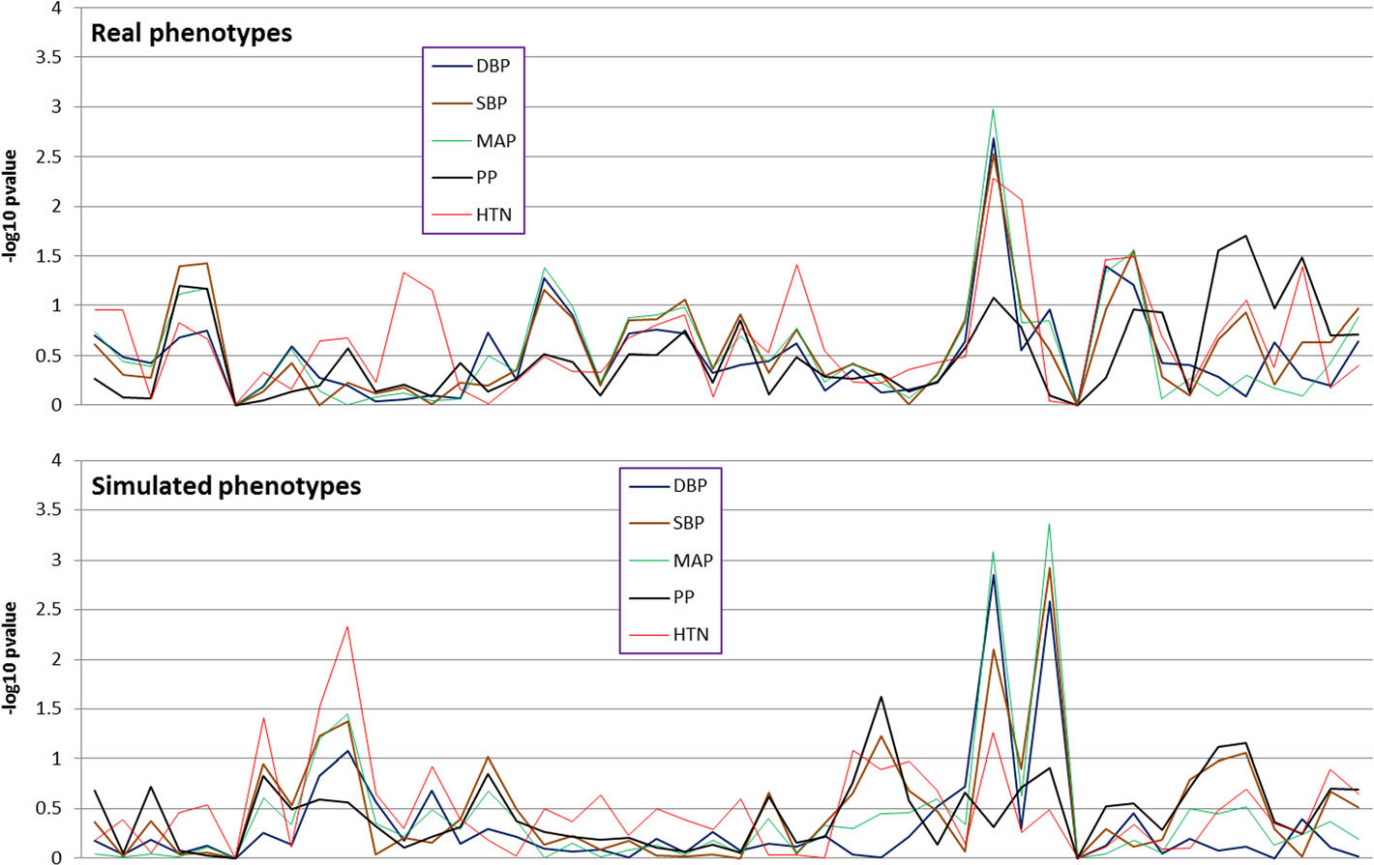
Distributions of haplotype association signals for real phenotypes *(top)* and simulated phenotypes *(bottom)*

Figure 1 displays the distributions of the single-variant association analysis for both real and simulated phenotypes. Table 2 shows the topmost pathway-based association signals. After Bonferroni correction for multiple testing, associations of *PRKCA* gene with DBP, SBP, and MAP remained significant in both real and simulated data. None of the empirical *p* values reached significant level. Figure 2 displays the distributions of the haplotype associations. The haplotype signals are similar to those of the single variant analysis in Fig. 1. Table 3 shows the results of the 2-locus epistasis analysis. The most significant interactions for real phenotypes were those between different genes, for example, *INS* vs. *PIK3R2* for DBP, whereas for simulated phenotypes there were significant within-genes interactions such as in *INSR* gene for SBP, MAP, and hypertension (Table 3).

**Table 2:** List of genes with top pathway-based signals for real and simulated phenotypes

Phenotype	Gene (variant)	*p* Value	
		Unadjusted	Bonferroni adjusted
Real phenotypes			
DBP	*PRKCA* (rs1010546)	0.0014	0.0291
SBP	*PRKCA* (rs1010546)	0.0015	0.0316
MAP	*PRKCA* (rs1010546	0.0006	0.0117
PP	*INSR* (rs3815902)	0.0130	0.2730
Hypertension	*PRKCA* (rs1010546)	0.0029	0.0624
Simulated phenotypes			
DBP	*PRKCA* (rs2227857)	0.0025	0.0528
SBP	*PRKCA* (rs2227857)	0.0009	0.0205
MAP	*PRKCA* (rs2227857)	0.0004	0.0080
PP	*INSR* (rs2860177)	0.0359	0.7549
Hypertension	*ATP1A4* (rs11265338)	0.0045	0.0953

**Table 3:** List of loci with most significant epistasis signals for real and simulated phenotypes

Phenotype	Locus 1	Locus 2	*p* Value
Real phenotypes			
DBP	*INS* (rs5506)	*PIK3R2 (rs1011320)*	0.0002169
SBP	*INSR* (rs3745548)	*PRKCG* (rs3745405)	0.0002839
MAP	*INSR* (rs3745548)	*PRKCG* (rs3745405)	0.0001629
PP	*PIK3CD* (rs28730674)	*PIK3R1* (rs61749601)	0.0000746
Hypertension	*PIK3CD* (rs11121484)	*ATP1B2* (rs1642763)	0.0004479
Simulated phenotypes			
DBP	*PIK3R1* (rs3730089)	*INSR* (rs78312382)	0.0001178
SBP	*INSR* (rs6413502)	*INSR* (rs3815902)	0.0000232
MAP	*INSR* (rs6413502)	*INSR* (rs3815902)	0.0002596
PP	*PIK3R3* (rs75775922)	*PIK3CA* (rs3729682)	0.0001188
Hypertension	*INSR* (rs2860177)	*INSR* (rs7252268)	0.0004135

## Discussion

In this study, we explored pathway-based analytical approach for the discovery of functional variants influencing blood phenotypes as additional method that could lead to identification of additional variants for complex human conditions. We focussed on a known biological pathway rather than pathways constructed from none proven biological systems. Our hypothesis was that because existing effective pharmacotherapeutic agents for blood pressure control act by targeting specific biological pathways, appropriate analytical methods that focus on such pathways could lead to identification of additional variants linked to complex human conditions than currently discovered by GWAS and candidate gene approaches. Results from this analysis indicate that, indeed, the use of known biological pathways for genetic association analysis can be a useful approach in the presence of true association since it takes advantage of the nonindependence of variants across pathway genes for setting threshold for statistical significance. We do note that because our analysis included genes from only the odd-numbered autosomes provided for GAW19, these results and their interpretations cannot be taken as fully representative of the aldosterone pathway. We are of the opinion that pathway-based analysis of variants from all genes in the pathway with those from the regulatory regions would lead to identification of important associations that can be interpreted with less limitation than in the present study. The use of known biological pathways in this study represents useful extension of genetic association analysis for complex human diseases.

## Conclusions

The findings from this study show that pathway-based analytical approaches can be useful in identification of important disease-associated variants that are otherwise undetectable by GWAS because of the assumption of nonindependence of variants within and across pathway genes which leads to reduced penalty of multiple testing and thus less stringent statistical significance threshold.
